# Unrepaired DNA damage in macrophages causes elevation of particulate matter- induced airway inflammatory response

**DOI:** 10.18632/aging.101412

**Published:** 2018-04-14

**Authors:** Man Luo, Zhengqiang Bao, Feng Xu, Xiaohui Wang, Fei Li, Wen Li, Zhihua Chen, Songmin Ying, Huahao Shen

**Affiliations:** 1 Key Laboratory of Respiratory Disease of Zhejiang Province, Department of Respiratory and Critical Care Medicine, Second Affiliated Hospital, Institute of Respiratory Diseases, Zhejiang University School of Medicine, Hangzhou 310009, China; 2 Cancer Centre, The Second Hospital of Shandong University, Jinan 250033, China; 3 Department of Pharmacology, Zhejiang University School of Medicine, Hangzhou 310058, China; 4 State Key Lab of Respiratory Disease, Guangzhou 510120, China

**Keywords:** DNA damage, DNA damage response, macrophages, particulate matter, airway inflammation

## Abstract

The inflammatory cascade can be initiated with the recognition of damaged DNA. Macrophages play an essential role in particulate matter (PM)-induced airway inflammation. In this study, we aim to explore the PM induced DNA damage response of macrophages and its function in airway inflammation. The DNA damage response and inflammatory response were assessed using bone marrow–derived macrophages following PM treatment and mouse model instilled intratracheally with PM. We found that PM induced significant DNA damage both *in vitro* and *in vivo* and simultaneously triggered a rapid DNA damage response, represented by nuclear RPA, 53BP1 and γH2AX foci formation. Genetic ablation or chemical inhibition of the DNA damage response sensor amplified the production of cytokines including Cxcl1, Cxcl2 and Ifn-γ after PM stimulation in bone marrow–derived macrophages. Similar to that seen *in vitro*, mice with myeloid-specific deletion of RAD50 showed higher levels of airway inflammation in response to the PM challenge, suggesting a protective role of DNA damage sensor during inflammation. These data demonstrate that PM exposure induces DNA damage and activation of DNA damage response sensor MRN complex in macrophages. Disruption of MRN complex lead to persistent, unrepaired DNA damage that causes elevated inflammatory response.

## Introduction

Airborne particulate matter (PM) pollution is a leading contributor to global disease burden [1]. Substantial epidemiological and clinical studies have documented that exposure to ambient air pollution is closely correlated with acute exacerbation of asthma and chronic obstructive pulmonary disease, respiratory tract infection and lung cancer incidence [2-5]. Therefore, it is of vital importance to determine the mechanisms responsible for pulmonary injury by PM and to develop potential therapy.

Multiple exogenous agents, such as ionizing radiation, ultraviolet light, thermal disruption and industrial chemicals, can cause a diverse spectrum of DNA lesions. To maintain genome stability, cells must initiate a prompt and correct response to DNA damage that is coordinated by a network of signal transduction pathways known as the DNA damage response (DDR). The cellular DDR system consists of sensors, signal transducers, mediators and effectors. As a DDR sensor, the MRE11-RAD50-NBS1 (MRN) protein complex plays diverse roles in the maintenance of genomic integrity, including repair of DNA double-strand breaks (DSBs), checkpoint activation, meiotic recombination and telomere maintenance [6-9]. Hypomorphic mutations in the MRN complex lead to Cancer-prone syndromes [10,11]. Furthermore, a RAD50-targeted treatment sensitizes squamous cell carcinoma to cisplatin-based chemotherapy, making possible the translation of the structural and functional exploration into a potentially applicable cancer therapy [12].

Accumulating evidence highlight direct links between DNA damage response and innate immune response signaling [13-20]. Persistent DNA damage signaling triggers increased secretion of inflammatory cytokines such as interleukin-6 (IL-6). Moreover, initiation and maintenance of this cytokine response requires the DDR proteins ATM, NBS1 and CHK2 [21]. However, it occurs in response to classical stimulation by exogenous insults such as viral or bacterial infections and endogenous byproducts of normal physiologic processes. Previous research has shown that airborne PM, a complex multi-component system threatening public health and safety, could cause relatively moderate damage to DNA in both *in-vitro* cell lines and occupational groups [22-24]. In this case, is there any connection between the DDR and inflammatory response induced by PM? Notably, alveolar macrophages (AMs) are the first responders to foreign particles in the lung and could phagocytose inhaled particles and produce a striking proinflammatory response upon exposure to PM [25,26]. Nevertheless, little is known about the possible role of DDR signaling in proinflammatory function of macrophages following PM exposure. Can PM cause direct DNA damage in macrophages? Is DDR signaling involved in PM-treated macrophages? Does DDR signaling impact the function of macrophages in PM-induced inflammation?

In this present study, we show that PM could trigger a direct DNA damage and subsequent DDR in macrophages. Using Genetic and chemical approaches, we further demonstrate that blocking DDR markedly enhances the PM-induced inflammatory response both *in vitro* and *in vivo*.

## RESULTS

### *In vivo* or *in vitro* exposure to PM induces DNA breaks in macrophages

**Figure 1:**
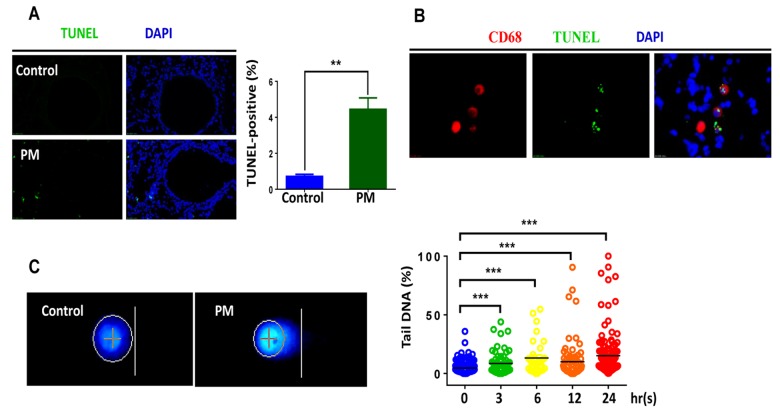
***In vivo* and *in vitro* exposure to particulate matter (PM) induces DNA breaks in macrophages.** Male C57BL/6 mice, aged 6-8 weeks (n=3) were instilled intratracheally with PM at 100 mg·d^-1^ for 2 days. After 24 hours lung tissues were collected, and DNA damage was analysed. (**A**) Lung tissues were stained with TUNEL (green) revealing the level of double-strand DNA breakage generated during apoptosis. The average percentage of TUNEL-positive cells to the total lung tissue cells with DAPI staining were quantified. (**B**) Representative immunofluorescence images of TUNEL (green) cells in alveolar macrophages are revealed using CD68 (red) staining. (**C**) Representative images of of alkaline comet assay in seven-day bone marrow–derived macrophages from wildtype mice stimulated with PM at 100 mg·mL^-1^ for time course (0h, 3h, 6h, 12h, 24h). Percentage of DNA intensity in the comet tail, was quantified. Each dot represents a single cell nucleus, and different colour represents corresponding exposure time(blue for 0h, green for 3h, yellow for 6h, orange for 12h, red for 24h) . The data are presented as means ± SEMs. *p < 0.05; **p < 0.01; ***p < 0.001.

Accumulating evidence suggests that various levels of PM exposure induce significant DNA damage in occupational populations and in cultured cell lines [27-29]. To further examine the genotoxic effects of PM on the airway epithelium or macrophages in vivo, male C57BL/6 mice, aged 6-8 weeks, were instilled intratracheally with PM at 100 μg·d^-1^·mouse^-1^ for 2 days. As revealed by TUNEL assay, the short-term PM exposure triggered remarkable DNA damage following apoptosis in the lung tissues (Fig. 1A). Surprisingly, DNA damage tended to be distributed in the peripheral pulmonary zone while the airway epithelium displayed inappreciable damage effects. Given that the TUNEL assay is limited in its sensitivity and specificity in detecting DNA damage following apoptosis [30], we used other assays in the following steps to further confirm the effects.

We next sought to confirm the locations of damage foci by marking alveolar macrophages (AMs) with CD68 staining (Fig. 1B). Double immunofluorescence labelling partially localized damage foci to AMs. These findings indicated that macrophages played important roles in PM-induced genotoxicity, at least during acute exposure. Consistent with *in vivo* findings, PM induced significant DNA damage in BMDMs, as revealed by the DNA percentage in the comet tail in a dose–dependent manner (Fig. 1C). In summary, these results clearly demonstrate that PM could induce DNA damage in macrophages both *in vivo* and *in vitro*.

### PM exposure induces DDR signaling in macrophages, involving upregulation of the RAD50 sensor protein

**Figure 2:**
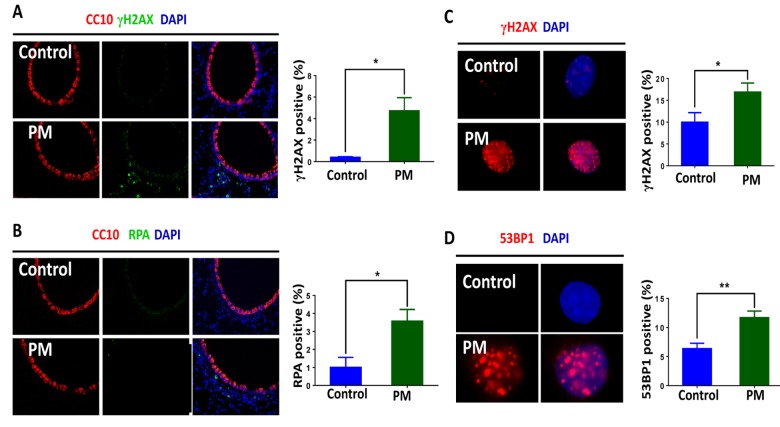
**Particulate matter (PM) exposure induces DNA damage response (DDR) signaling in macrophages.** C57/BL6 mice (n=3) were instilled intratracheally with PM at 100 μg·d^-1^ for 2 days *in vivo*. After 24 hours lung tissues were collected, and DNA damage response was analysed. (**A**) Representative image of γH2AX (red) in lung tissues. The average percentage of γH2AX positive cells to the total lung tissue cells with DAPI staining were quantified. (**B**) Analysis of RPA (red) in lung tissues. The average percentage of RPA positive cells to the total lung tissue cells with DAPI staining were quantified. (**C**) Representative immunofluorescence images of γH2AX (red) in bone marrow–derived macrophages (BMDMs) stimulated with PM at 100 μg·mL_-1_ for 24 hours. Cells with 5 or more foci were counted as positive. (**D**) Analysis of 53BP1 (red) in BMDMs treated with PM at 100 μg·mL^-1^ for 24 hours. Cells with 5 or more foci were counted as positive. All experiments were repeated at least 3 times, and data are presented as means ± SEMs. *p < 0.05; **p < 0.01; ***p < 0.001.

**Figure 3:**
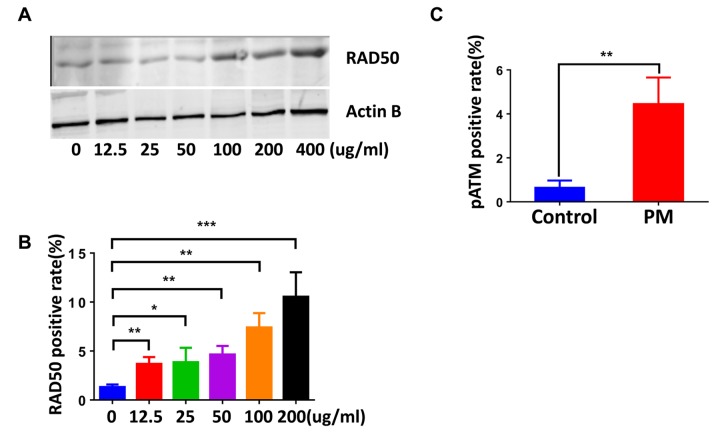
**The DNA damage sensor, RAD50, is induced in particulate matter (PM)-stimulated macrophages.** (**A**) Bone marrow–derived macrophages (BMDMs) were treated with PM (100 μg·mL^-1^) for 24 hours, then were stained with RAD50. Quantification of RAD50-positive cells for a dose response of PM. (**B**) Western blot analysis for a dose response (various concentrations of PM for 24 hours) of RAD50 protein expression in BMDMs treated with PM. (**C**) Quantification of pATM-positive cells in BMDMs treated with PM at 100 μg·mL^-1^ for 24 hours. All experiments were repeated at least 3 times, and data are presented as means ± SEMs. *p < 0.05; **p < 0.01; ***p < 0.001.

We next examined DDR signaling in the lung tissues and BMDMs. Similar to that seen where the DNA damage occurred, phosphorylated histone H2AX(γH2AX) and RPA foci, DDR markers, primarily occurred in peripheral lung tissue rather than the airways. Quantification of γH2AX and RPA also demonstrated a marked elevation of DDR in response to PM exposure. (Fig. 2A and B). Likewise, quantification of γH2AX-positive cells (≥5 foci per nucleus) revealed higher levels of DNA damage in BMDMs upon PM stimulation (Fig. 2C). PM exposure also slightly increased the frequency of 53BP1-positive cells (≥5 foci per nucleus) in BMDMs (Fig. 2D). Because these events require rapid recognition of the DDR sensor, we next examined the expression patterns of RAD50, a subunit of the MRN complex, using immunofluorescence and western blots. As shown in (Fig. 3A and B), RAD50 protein levels increased with PM exposure in a dose-dependent manner. Because phosphorylation of H2AX requires activation of the phosphatidylinositol-3-OH-kinase-like entities ATM and ATR kinases, we next determined whether the observed increase in γH2AX was related to the autophosphorylation of ATM. As shown in (Fig. 3C), immunofluorescence staining demonstrated an increase in phosphorylated ATM protein at serine 1981 in BMDMs upon PM exposure.

### Genetic deletion of RAD50 in macrophages increases cytokine production following PM stimulation

**Figure 4:**
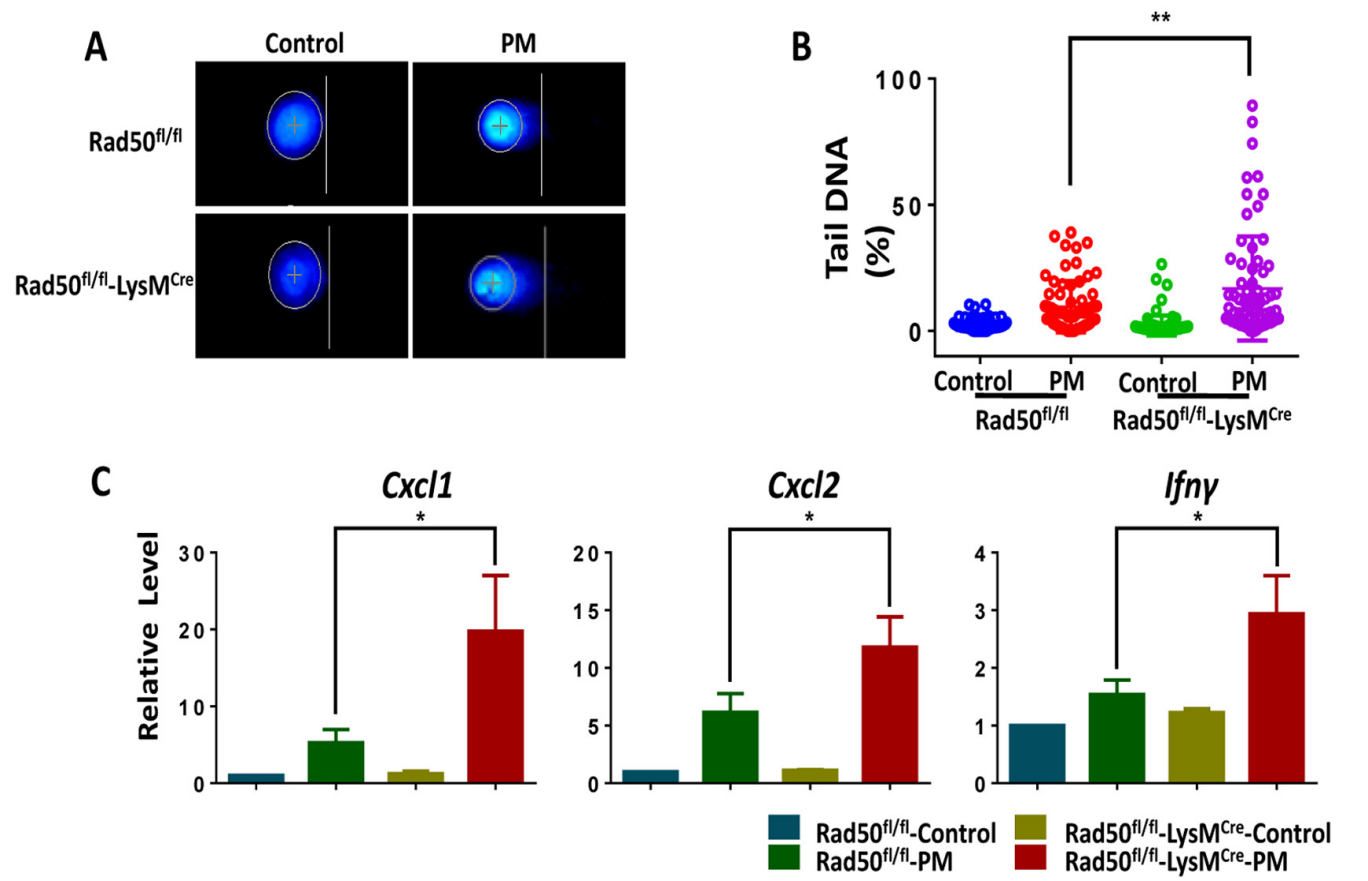
**Genetic deletion of RAD50 in macrophages increases cytokine production following particulate matter (PM) stimulation.** BMDMs from mice with RAD50 deletion in myeloid cells (RAD50^flox/flox^-LysM^cre^) and wildtype (WT) mice (RAD50^flox/flox^) were treated with PM (100 μg·mL^-1^). (**A**) BMDMs were stimulated with PM for 3 hours. Representative images of alkaline comets. (**B**) Quantification of DNA damage. (**C**) BMDMs were stimulated with PM for 24 hours. Relative levels of Cxcl1, Cxcl2 and Ifn-γ were determined using quantitative PCR. Data are presented as means ± SEMs across at least 3 independent experiments. *p < 0.05; **p < 0.01.

Our findings suggest that the DDR sensor RAD50 could have important functionalities. According to previous studies [18,31,32], we hypothesized that the absence of a DDR sensor in mice could lead to persistent, unrepaired DNA damage, and thus augments fine PM-induced inflammatory cytokine secretion. To corroborate hypothesis, we used BMDMs from mice with RAD50 deletion in myeloid cells (RAD50^flox/flox^-LysM^cre^) and wildtype (WT) mice (RAD50^flox/flox^). To test this idea, we first checked direct DNA damage using the alkaline comet assay. The BMDMs from RAD50^flox/flox^-LysM^cre^ mice displayed relatively high basal levels of DNA damage, which was further increased after PM exposure, contrasting with results found with WT controls (Fig. 4A and B). We next detected the inflammatory response. Treatment with PM induced significantly higher levels of Cxcl1, Cxcl2 and Ifn-γ in RAD50^flox/flox^-LysM^Cre^ macrophages compared with those in WT cells, as determined using real-time PCR (Fig. 4C). In summary, loss of DDR sensor RAD50 in macrophages augments DNA breaks, resulting in amplified inflammatory cytokine response *in vitro*.

### Chemical inhibition of Mre11, a central MRN complext component, promotes PM-induced cytokine production

**Figure 5:**
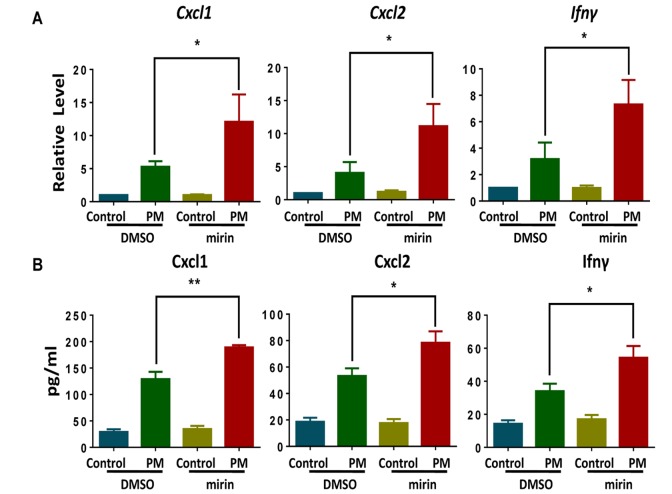
**Chemical inhibition of Mre11, a central MRE11-RAD50-NBS1 (MRN) complex component, promotes particulate matter (PM)-induced cytokine production.** Wildtype bone marrow–derived macrophages (BMDMs) were incubated with and without the MRN complex inhibitor mirin (administered dose of 100 μM) for 2 hours, then were treated with PM (100 μg·mL^-1^) for an additional 24 hours. (**A**) The relative levels of Cxcl1, Cxcl2 and Ifn-γ mRNA transcripts were determined using quantitative PCR. (**B**) The protein levels of Cxcl1, Cxcl2 and Ifn-γ in the culture supernatants were measured using ELISA. Data are presented as means ± SEMs across at least 3 independent experiments. *p < 0.05; **p < 0.01.

To further confirm the role of the DDR sensor in the inflammatory response in macrophages, we used mirin, a potent MRN complex inhibitor [33], to block the MRN complex by inhibiting Mre11-associated exonuclease activity. Compared with the control group, BMDMs exposed to PM after treatment with mirin (administered dose of 100 μM) produced marked elevations in both mRNA and cytokine protein levels, including Cxcl1, Cxcl2 and Ifn-γ (Fig.5A and B). These results strengthen the idea that the disruption of MRN complex causes elevated inflammatory response in BMDMs upon PM exposure.

### RAD50^fl/fl^-LysM^Cre^ mice display exacerbated airway inflammation in response to PM exposure

**Figure 6:**
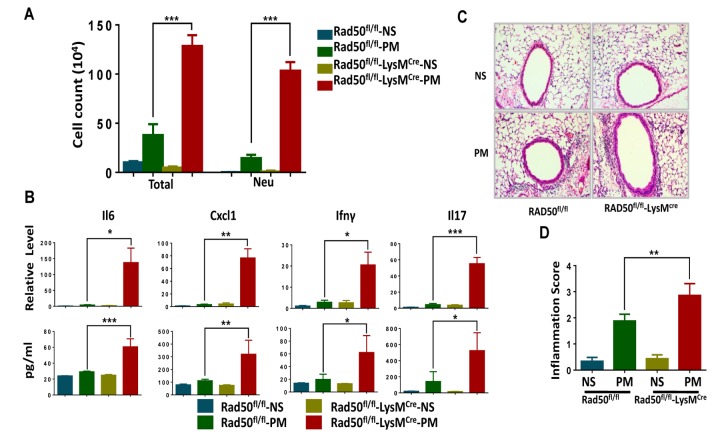
**RAD50^flox/flox^-Lys^Mcre^ mice display exacerbated airway inflammation in response to particulate matter (PM) exposure.** RAD50^flox/flox^-LysM^cre^ male mice and their wildtype littermates (n =5, 6, or 7 per group) were instilled intratracheally with PM at 100 μg·d-1 or the equivalent volume of normal saline (NS) as control for 2 days, and after 24 hours. In the bronchoalveolar lavage fluid, (**A**) the total number of inflammatory cells was quantified and the number of neutrophils was calculated. (**B**) Expressions of Il6, Cxcl1, Ifn-γ and Il17 levels in the lung tissue were determined using real-time PCR and ELISA. (**C**) Representative images of lung sections stained with hematoxylin and eosin (H & E). (**D**) Semiquantified inflammation score of the H & E staining (n =10 images per group). *p < 0.05, **p < 0.01,***p < 0.001.

To further elucidate the role of the DDR sensor in airway inflammation *in vivo*, we challenged RAD50^flox/flox^ -LysM^cre^ mice and their WT littermates with PM or normal saline(NS) via intratracheal instillation. The total number of inflammatory cells and neutrophils in the bronchoalveolar lavage fluid from RAD50^flox/flox^-LysM^cre^ mice was significantly higher relative to the littermate controls (Fig. 6A). Analysis of lung extracts revealed increased mRNA and protein levels of cytokines such as Cxcl1, Il6, Il17 and Ifn-γ in RAD50^flox/flox^-LysM^cre^ mice following treatment with PM (Fig. 6B). Furthermore, PM-induced airway inflammation was significantly upregulated in RAD50^flox/flox^-LysM^cre^ mice, and this was confirmed using immunohistological analysis (Fig. 6C and D). These results demonstrate that RAD50^flox/flox^-LysM*^cre^* mice with impaired DNA damage sensor display exacerbated proinflammatory responses.

## DISCUSSION

In this present study, we found that DDR signaling play an important role in PM-induced inflammatory responses in macrophages. We have shown that PM can trigger direct DNA damage and subsequent DDR signaling in macrophages both *in vitro* and *in vivo* and that genetic ablation or chemical inhibition of DDR augments PM-induced cytokine production in BMDMs. Meanwhile, myeloid-specific deletion of the DDR–related gene RAD50 exacerbates airway inflammation in response to PM exposure *in vivo*.

Substantial evidence shows that PM induces DNA damage in a variety of lung cells such as epithelium and alveolar macrophages [22,28,29,34,35]. Moreover, it triggers damage in the peripheral lung region where the vast majority of the inhaled particles are deposited [36,37]. The evidence shows that PM leads to the induction of DNA lesions in alveolar macrophages while, interestingly, the airway epithelium displays slight or undetectable levels of DNA damage, as shown by TUNEL assay combined with the immunofluorescence of phosphorylated histone H2AX(γH2AX) and RPA (Fig. 1A, 2A). Macrophages are particularly damaged, primarily because they are the first responders and engulf inhaled foreign matter in the lungs upon PM exposure. It should be noted that many epidemiological and clinical studies reveal the adverse effects of exposure to air pollution via big data analysis, and study participants have undergone long-term exposure [2,3,5,38,39]. In this study, we examined the genotoxic effects of PM in a short-term model, and this difference could explain why the DNA damage we found in macrophages upon stimulating with PM was modest.

Given that DNA experiences a wide range of lesions, repair mechanisms specific for many types of lesions have evolved. Single-strand DNA breaks are repaired by single-strand break repair (SSBR), while DNA double-strand breaks (DSBs) are repaired by two main pathways: the non-homologous end joining pathway (NHEJ) or homologous recombination (HR), with chromatin motion during repair [40,41]. In response to PM10-induced genotoxic stress, A549 cells showed activation of the ATM/ATR/Chk1/Chk2/p53 pathway to repair the DNA lesions [22]. Recently, PM2.5 has reportedly induced ATR, rather than ATM to regulate TP53-dependent autophagy induction in Beas-2B cells [29]. In this study, phosphorylated histone H2AX (γH2AX), which attaches to chromatin near DSBs and induces the repair of damaged DNA [42], demonstrated a marked elevation upon PM exposure both in lung tissues and BMDMs. In addition, RPA and 53BP1 showed an increased foci formation in the mouse model and in BMDMs, respectively (Fig. 2B and D). Phosphorylated ATM protein, consistent with the literature, is significantly increased following PM treatment (Fig. 3C). Furthermore, formation of PM-induced DNA strand breaks showed no obvious time dependence, and only a few cell deaths were seen after additional time was given (Fig. 1C). These findings reveal that DDR signaling was initiated in response to DNA damage, and most of the damaged sites were properly repaired.

A growing body of literature suggests that DDR signaling is critical for an immune response (e.g., [43]). ATM plays an essential role in redox balance, which contributes to the notable susceptibility of patients with ataxia-telangiectasia (AT) to bacterial infections [20]. Neutrophils with mutations in the ATM overproduce proinflammatory cytokines IL-8, and activation of ATM and DNA damage signaling suppress cytokine production [44]. The MRN complex has also been found to be crucial in regulating inflammation. Both MRE11 and RAD50 induce type I interferon by regulating STING trafficking [32]. Meanwhile, NBS1, a component of the MRN complex, is crucial for macrophage function during normal aging, and depletion of NBS1 in macrophages show enhanced inflammatory responses in a 2,4-dinitrofluorobenezene (DNFB) irritation model [31]. In this study, we confirmed the role of the MRN complex, a DDR sensor, in the macrophages’ proinflammatory function using RAD50^flox/flox^-LysM^cre^ mice and corresponding BMDMs upon PM stimulation. These findings, together with chemical inhibition *in vitro*, imply that the essential role of DDR sensor in macrophage in preserving the appropriate level of PM-induced airway inflammation.

A limitation of our study is that further research is required to investigate the mechanism of how unrepaired DNA damage resulting in disruption of DNA damage response mediate PM-induced inflammatory responses in macrophages. DNA damage has been shown to prime the type I IFN system to promote antimicrobial responses via the STING pathway [18], while the DNA damage sensor Rad50 translocates to the cytoplasm, forming dsDNARad50-CARD9 complexes, and selectively induces NF-κB signaling for IL-1β production [17]. Most recently, micronuclei generated at mitosis have been promoted to activate the cGAS–STING pathway, which links genome instability to innate immune responses [45]. Based on these studies, we are now working on the interaction between DNA damage response and micronuclei and downstream cGAS–STING pathway in BMDMs and macrophages cell line RAW264.7.

## CONCLUSIONS

In summary, this study demonstrates that PM exposure induces DNA damage and subsequently initiates DNA damage response in macrophages. Disruption of MRN complex causes elevated inflammatory response through accumulated unrepaired DNA damage.

## MATERIALS AND METHODS

### Mice

Mice were maintained on a mixed C57BL/6 and 129Sv background. Myeloid cell-specific RAD50 conditional knockdown mice (RAD50^flox/flox^-LysM^cre^) were obtained by crossing RAD50^flox/flox^ mice with mice expressing Cre recombinase under the control of the Lysozyme promoter (LysM^cre^). LysM^cre^-negative, RAD50^flox/flox^ littermates served as controls. The RAD50^flox/flox^ mice were obtained from Jackson Laboratory, and the LysM^cre^ mice on the C57BL/6 background were a generous gift from Dr. Gen-Sheng Fen (University of California at San Diego, CA, USA). The WT C57BL/6 mice were purchased from the Laboratory Animal Center of Zhejiang University (Hangzhou, China). Four- to five-week-old mice were typically used for *in vitro* experiments. Six- to eight-week-old mice were used for *in vivo* studies. All mice were maintained in a specific-pathogen-free facility. All experimental protocols were approved by the Ethical Committee for Animal Studies at Zhejiang University.

### Cell preparation

Bone marrow was harvested and cultured for 6 days in complete Dulbecco's Modified Eagle Medium containing 10% heat-inactivated FBS (Gibco 10099-141), 1% penicillin-streptomycin (Gibco, 15140-122) and 10 ng·mL^-1^ mouse M-CSF (R&D Systems, 416-ML-010). On day 6, a homogeneous population of adherent macrophages was obtained after 6 days of culture (>95% CD11b and F4/80). In a subset of experiments, BMDMs were treated with PMs at indicated concentration and time points. In experiments in which MRN complex activity was blocked chemically, mirin (100 nM; Selleck) was used.

### *In vitro* and *in vivo* PM exposure

Ambient urban PM, Standard Reference Material 1649b, was obtained from the National Institutes of Technology (Gaithersburg, MD, USA). The PM was suspended and sonicated in sterile saline at a concentration of 2 mg·mL^-1^ (mass/volume). *In vitro*, BMDMs were treated with PMs at 100 μg·mL^-1^. *In vivo*, mice were instilled intratracheally with PM 100 μg (in 50 μL saline) per day for 2 days.

### RNA isolation and quantitative real-time PCR analysis

From cells and lung tissues, RNA was isolated using Trizol (Takara Biotechnology). Reverse transcription was performed using reverse transcription reagents (Takara Biotechnology). The expression of mouse Cxcl1, Cxcl2 and Ifn-γ were measured using quantitative real-time PCR with SYBR Green Master Mix (Takara Biotechnology) on a StepOne real-time PCR system (Applied Biosystems, Foster City, CA, USA). All protocols were performed according to manufacturer’s instructions. Primer sequences used are as follows:

Actin B:

Forward: 5′-GTCCACCGTGTATGCCTTCT-3′,

Reverse: 5′-CTCCTGGTGTCCGAACTGAT-3′;

Cxcl1:

Forward: 5’-CTGGGATTCACCTCAAGAACATC-3’,

Reverse r: 5’-CAGGGTCAAGGCAAGCCTC-3’;

Cxcl2:

Forward: 5’-TGTCCCTCAACGGAAGAACC-3’,

Reverse: 5’-CTCAGACAGCGAGGCACATC-3’;

Il6:

Forward: 5’-CTGCAAGAGACTTCCATCCAG-3’,

Reverse: 5’-AGTGGTATAGACAGGTCTGTTGG-3’;

Ifnγ:

Forward: 5’-ACAGCAAGGCGAAAAAGGATG-3’,

Reverse: 5’-TGGTGGACCACTCGGATGA-3’;

Il17:

Forward: 5’-TCAGCGTGTCCAAACACTGAG-3’,

Reverse: 5’-CGCCAAGGGAGTTAAAGACTT-3’;

### Chemicals and reagents

Antibodies against CC10 (Santa Cruz Biotechnology, sc-9772), CD68(Servicebio, GB11067), Actin B (Santa Cruz Biotechnology, sc-47778), RAD50(Abcam, ab89), γH2AX (EMD Millipore, 05-636), RPA (Abcam, ab2175), pATM (EMD Millipore) and 53BP1 (EMD Millipore, MAB3802) were used. Antibodies for immunofluorescence were diluted at 1:1000 in 5% BSA (Sigma-Aldrich, B2064) except for the pATM which was diluted at 1:500. Antibodies for western blot were diluted at 1:1000 in 5% nonfat dried milk. All primers used in the study were synthesized by Sangon Biotech,Shanghai. ELISA kits for mouse Cxcl1 (MKC00B), mouse Cxcl2 (MM200), mouse Il6 (M6000B), mouse Il17(R&D, M1700) and mouse Ifn-γ (R&D, MIF00) were purchased from R&D systems. MRE11 inhibitor mirin was purchased from Selleck (S8096), and its final concentration in culture medium was 100μM.

### ELISA

The concentrations of Cxcl1, Cxcl2, Il6, Il17and Ifn-γ in cultured medium and bronchoalveolar lavage fluid supernatants were determined using ELISA kits from R&D Systems following manufacturer’s instructions.

### Immunofluorescence

Bone marrow–derived macrophages were seeded on cleaned and autoclaved glass coverslips. Following the indicated treatment, the cells were washed with phosphate-buffered saline (PBS) and then fixed in 4% paraformaldehyde solution for 30 min at room temperature. Subsequently, the cells were permeabilized with 0.1% Triton-X-100 (Sigma-Aldrich, LC262801) in PBS for 10 min at 4°C and blocked with 5% BSA (Sigma-Aldrich, B2064) for 30 min at room temperature followed by washing with PBS. Then the cells were incubated with primary antibody overnight at 4°C. After washing the cells with PBS, Alexa Fluor®555-conjugated goat anti-mouse IgG (HCL) antibody (Invitrogen, A28180) solution was added and incubated for 1 h at room temperature, washed with PBS. All DNA were counterstained with DAPI. Fluorescent images were captured with a Zeiss LSM laser scanning confocal microscope. For each condition, 10 fields of at least 14 cells each were quantified, and cells with 5 or more foci were counted as positive.

For the double immunofluorescence labeling, cells were fixed, permeabilized and blocked as previously described. Then incubate cells in the mixture of two primary antibodies (e.g.goat against CC10 and mouse against γH2AX or RPA) in 5% BSA in PBST in a humidified chamber overnight at 4°C. Wash the cells three times in PBS, 5 mins each wash. Incubate cells with the mixture of two secondary antibodies which are raised in different species (with two different fluorochromes, e.g. Alexa Fluor®488-conjugated goat anti-mouse IgG (HCL) antibody and Cy3-conjugated donkey Anti-goat IgG (H+L)) in 5% BSA for 1 hr at room temperature in dark. DNA was counterstained with DAPI. Finally the samples were analyzed under fluorescence microscope as described above.

### Comet assay

Bone marrow–derived macrophages were cultured as described. After incubation, cells were harvested, processed and analyzed using a Comet Assay Kit (Trevigen) according to manufacturer's protocol. After assay, slides were stained with DAPI and viewed by epifluorescence microscopy. Cell images were analyzed using CASP software (downloaded from www.casplab.com). Among the comet parameters, we report the percent DNA in the tail (tail DNA%) as a marker of DNA damage. Two hundred comets were scored randomly for each indicated treatment. All experiments were performed in triplicate.

### TUNEL assay

The TUNEL (terminal deoxynucleotidyl transferasemediated dUTP nick end labeling) assay was performed to detect DNA fragmentation in bone marrow–derived macrophages by using a commercial kit (Roche Diagnostics Limited, In situ Cell Death Detection kit, fluorescein, 11684795910). Paraffin-embedded lung tissue sections were permeabilized in 0.1% Triton X-100(Sigma-Aldrich, LC262801), blocked nonspecific binding with 5% bovine serum albumin (BSA) in PBS for 1h at room and then incubated with TUNEL reaction mixture containing label solution and enzyme solution in a humidified atmosphere for 60min at 37°C. Nuclei were counterstained with DAPI. Samples were analyzed under fluorescence microscope for DNA breaks. The TUNEL positive rate was determined according to the formula: (number of cell with green staining/total number cells with DAPI staining) × 100%.

To perform a TUNEL assay with CD68 co-staining, Lung tissues were permeabilized and blocked as described previously. Incubate coverslips in a humidified chamber with CD68 (dilution at 1:1000 in 5% BSA) overnight at 4°C. incubated with 50μL TUNEL reaction mixture in a humidified atmosphere for 60min at 37°C following wash with PBS. and then 50μL of 555-conjugated goat anti-rabbit IgG (HCL) for 1h at 37°C. Rinse coverslips 3× 10min in PBS. DNA was counterstained with DAPI. Analyze coverslips under fluorescence microscope as described above.

### Western blot

Whole-cell extracts were prepared by lysis and sonication of cells in RIPA buffer (Beyotime, P0013B) containing phosphatase inhibitors (Roche Diagnostics GmbH, 04-906-837-001) and protease (Roche Diagnostics GmbH, 04-693-116-001). Lysates were run on SDS–PAGE, then transferred onto PVDFmembrane (millipore, IPVH00010), imunoblotted with relevant antibodies using standard methods. Actin B served as a protein loading control.

### Histological analysis

Following each indicated treatment, lung tissues were fixed and stained with hematoxylin and eosin to demonstrate general morphology. Slides were digitized using an Olympus BX53 inverted microscope (Olympus, Melville, NY). The severity of inflammation was graded semiquantitatively according to published guidelines [46] as follows: 0, normal; 1, few cells; 2, a ring of inflammatory cells 1 cell layer deep; 3, a ring of inflammatory cells 2–4 cells deep; 4, a ring of inflammatory cells of >4 cells deep.

### Statistical analysis

Statistical significance was calculated using the two-tailed Student *t* test unless otherwise indicated. Prism 6 software was used to generate graphs and statistical analyses. Statistical significance was recognized when p ≤ 0.05.
